# Adverse Events in Osimertinib Treatment for EGFR-Mutated Non-Small-Cell Lung Cancer: Unveiling Rare Life-Threatening Myelosuppression

**DOI:** 10.3390/medicina60081270

**Published:** 2024-08-06

**Authors:** Walid Shalata, Ashraf Abu Jama, Yulia Dudnik, Omar Abu Saleh, Sondos Shalata, Lena Tourkey, Kim Sheva, Amichay Meirovitz, Alexander Yakobson

**Affiliations:** 1 The Legacy Heritage Cancer Center and Dr. Larry Norton Institute, Soroka Medical Center, Beer Sheva 84105, Israel; 2 Faculty of Health Sciences, Ben-Gurion University of the Negev, Beer Sheva 84105, Israel; 3 Department of Dermatology and Venereology, Emek Medical Centre, Afula 18341, Israel; 4 Nutrition Unit, Galilee Medical Center, Nahariya 22000, Israel

**Keywords:** adverse events, osimertinib, tagrisso, targeted therapy, EGFR mutation, non-small-cell lung cancer, myelosuppression

## Abstract

Recent advancements in targeted therapies for non-small-cell lung cancer (NSCLC), specifically focusing on epidermal growth factor receptor (EGFR) mutations, have revolutionized treatment strategies. Osimertinib, an approved therapy for metastatic NSCLC with EGFR mutations, highlights remarkable efficacy but also harbors the potential for severe adverse events, whose rarity or lack of precedence may mask their criticality. This article delves into the exploration of adverse events linked to osimertinib, shedding light on a rare yet life-threatening occurrence: severe myelosuppression. A case study detailing a patient with EGFR-mutated NSCLC exhibiting a robust treatment response but experiencing severe myelosuppression following osimertinib initiation is presented. Immediate discontinuation of osimertinib alongside concurrent blood transfusions facilitated toxicity recovery, prompting a successful reduction in myelosuppression severity upon re-administration at a lowered dosage.

## 1. Introduction

Lung cancer is responsible for causing the highest number of cancer-related deaths worldwide, where, in 2017, this cancer type caused more deaths than breast, prostate, colorectal, and brain cancers combined [1,2]. More than 80% of lung cancer cases are classified as non-small-cell lung cancer (NSCLC), with this cancer subtype being one of the main causes of the high lung cancer death rate [3,4]. NSCLC presents with few, if any, symptoms in its initial phases, resulting in most patients seeking treatment only once the disease has advanced and metastasized. This contributes significantly to a dismal patient prognosis [5].

When lung cancer is found to be mutated in the epidermal growth factor receptor (EGFR), targeted drugs of the tyrosine kinase inhibitor (TKI) family have proven to be successful treatment options. Multiple clinical studies have shown the superiority of EGFR TKIs as a first-line treatment [6,7,8] over chemotherapy, with a response rate of 70% [9]. There are currently five TKI drugs categorized into three generations that are available to treat EGFR-mutated NSCLC. First- and second-generation TKIs include erlotinib and gefitinib, and afatinib and dacomitinib, respectively, where osimertinib is a third-generation TKI. Osimertinib is the only agent with FDA approval to be utilized as adjuvant treatment for EGFR-mutated NSCLC [10].

Osimertinib, an irreversible, orally administered EGFR-TKI, was initially approved by the FDA in 2015 and was intended for use only as an adjuvant therapy after tumor resection in adult NSCLC patients with EGFR exon 19 deletions or exon 21 L858 mutations. In 2018, indications for osimertinib were expanded for use as a first-line therapy in adults with metastatic NSCLC whose tumors bare the aforementioned mutations. Osimertinib is also indicated for the treatment of metastatic NSCLC patients who test positive for the EGFR T790M mutation and whose disease has progressed during or following EGFR TKI therapy. Osimertinib covalently binds to certain mutant forms of EGFR, i.e., exon 19 deletion, L858R, and T790M, to prevent the activation of the EGFR-mediated cell signaling cascade. In this way, the drug works as an antagonist with antitumoral capabilities to potentially inhibit neoplasm growth in EGFR-overexpressing tumor cells and induce cell death [11,12].

In the FLAURA study for patients treated with osimertinib, several adverse events were reported. The most common side-effects included diarrhea (60%), rash or acne (59%), and nail effects (39%). Dry skin and stomatitis were also notable, affecting 38% and 29% of patients, respectively. Other frequently observed side-effects included decreased appetite (24%), cough (22%), and nausea (20%). Constipation, pruritus, and renal symptoms were reported in 18% of patients each. Fatigue and anemia affected 16% of patients, while dyspnea and vomiting were reported in 15% of patients. Headache, back pain, and upper respiratory tract infections occurred in 13% of patients. Pyrexia, insomnia, and nasopharyngitis were noted in 11% of patients each. Less common but significant effects included prolonged QT interval, increased aspartate aminotransferase, musculoskeletal pain, and alopecia [13].

Herein, we describe the first documented case of severe myelosuppression in a patient with metastatic non-small-cell lung cancer (NSCLC) harboring EGFR mutations. This life-threatening case condition occurred as a result of osimertinib treatment.

## 2. Case Report

In January 2020, a 60-year-old female referred to the emergency room of Soroka Medical Center due to a rash on her hands and face, accompanied by itching and weakness. She was an otherwise healthy non-smoker on no medication and with no family history of cancer. She was hospitalized for further investigations. Laboratory investigations, including a complete blood count, biochemical profile, rheumatologic tests, oncologic markers, tuberculosis tests, urine tests, and stool tests, were within the normal range. A multidisciplinary conference, including a dermatologist, rheumatologist, and neurologist, concluded that the patient presented with a clinical picture matching that of dermatomyositis. Therefore, a skin biopsy was performed where histopathological results supported a diagnosis of dermatomyositis. Chest radiography (CXR) showed a rounded opacity in the left upper lobe (LUL). For further investigation, the patient underwent a total body computed tomography scan (CT) that visualized an LUL mass with a diameter of 17 mm, as well as enlarged neck and mediastinal lymph nodes (left). Magnetic resonance imaging (MRI) of the head showed no evidence of brain metastases. A left supraclavicular lymph node biopsy confirmed metastatic adenocarcinoma of the lung (poorly differentiated; TTF-1-positive; P40-negative). Positron emission tomography–computed tomography (PET-CT) showed hyper-metabolic uptake in the LUL mass (diameter of 17 mm) and the neck and mediastinal lymph nodes (11 mm), as well as in a splenic mass (diameter of 12 mm). A clinical diagnosis of metastatic non-small-cell lung cancer (NSCLC) T1N3M1 stage IV was made. Further molecular analysis revealed the disease to be EGFR-positive (p.Glu746_Ser752delinsVal.), PDL1 > 50%, ROS1-negative, ALK-negative, and BRAF-negative.

Treatment with osimertinib was initiated in February 2020 at a standard dose of 80 mg once daily. A complete blood count was performed and was within the normal range at this time. A month later, the patient’s dermatological symptoms were completely diapered, and we came to the conclusion that the clinical picture of dermatomyositis was paraneoplastic syndrome; two months later, the patient underwent a follow-up PET-CT, which revealed a good response to treatment with relatively less uptake in the LUL mass, and treatment with osimertinib was continued. In December 2020, MRI and PET-CT follow-ups showed stable disease; however, due to arthralgia, fatigue, and weakness, a complete blood count was performed that revealed moderate anemia (9 gr/dL (normal 12–16)), leukopenia (1.83 × 10^3^/uL (normal 4.8–10.8)), neutropenia (1.72 × 10^3^/uL (normal 2–8 × 10^3^/uL)), and moderate thrombocytopenia (76 × 10^3^/uL (normal 130–140)). Ten months later (October 2021), the patient was admitted to the emergency department upon being found comatose at home. Blood tests revealed severe myelosuppression (anemia of 4.3 gr/dL, thrombocytopenia of 5 × 10^3^/uL, leukopenia of 0.92 × 10^3^/uL, and neutropenia of 0.52 × 10^3^/uL (normal 2–8 × 10^3^/uL)); the patient was therefore admitted into the intensive care unit (I.C.U). A bone marrow biopsy was performed that showed no metastasis or malignancy, with no other pathological findings present. Osimertinib treatment was suspended, the patient was administered platelets and erythrocytes, and prednisone treatment was initiated. After one week, the patient’s condition had improved significantly, osimertinib treatment was resumed as before, and she was sent home. By December 2021, upon weekly follow-up blood tests, the patient’s condition had improved and her complete blood count returned to an acceptable range with hemoglobin (10.1 gr/dL), neutrophils (3.2 × 10^3^/uL), and platelets (46 K × 10^3^/uL). The patient had mild myelosuppression that did not require medical intervention; she was asked to continue follow-ups for blood tests. During this period, we communicated with “AstraZeneca” who initially indicated no connection to the treatment, and the patient continued rigorous follow-ups with blood tests. Two months later (February 2022), however, the patient was again admitted to the I.C.U in a comatose state with a complete blood count revealing the return of severe myelosuppression with hemoglobin (6.2 gr/dL), leukopenia (1.2 × 10^3^/uL), neutrophils (0.94 × 10^3^/uL), and platelets (10 K × 10^3^/uL). Osimertinib treatment was immediately suspended. Blood and platelet transfusions were performed. A bone marrow biopsy was performed that revealed no evidence of metastatic carcinoma or plasma cell neoplasm or lymphoma.

The myelosuppression was suspected to be a drug-related adverse effect and thus, upon release from the hospital, the patient was carefully monitored by weekly blood tests, while osimertinib treatment was resumed as before (80 mg daily). The weekly blood tests began to show the progressive return of myelosuppression. The osimertinib dosage was thus reduced to 80 mg every alternative day. The rate of progression of the myelosuppression decreased; however, it continued to worsen and, as a result, the osimertinib dose was decreased to 40 mg every second day.

In April 2022, the patient’s blood count showed an improvement in hemoglobin (10 gr/dL), leukopenia (1.2 × 10^3^/uL), neutrophil (3.5 × 10^3^/uL), and platelet (36 K × 10^3^/uL) counts. We continued to monitor our patient with weekly blood tests, which consistently showed stability. This approach effectively reduced myelosuppression to Grade 1/2, with no further events of severe myelosuppression observed. At present (July 2024), the patient remains on osimertinib treatment at 40 mg every second day with grade 1/2 myelosuppression that is treated with blood transfusions as needed.

## 3. Discussion

Lung cancer is the most commonly occurring cancer globally with NSCLC accounting for the vast majority of cases and EGFR being one of the main driver genes [1,14]. Multiple randomized phase III studies found both first- and second-generation EGFR-TKIs, gefitinib, erlotinib, and afatinib to significantly improve overall survival in advanced EGFR-mutated NSCLC as compared to standard, platinum-based combination chemotherapy [15,16]. This resulted in EGFR-TKI being recommended as the first-line treatment for patients with advanced EGFR-mutated NSCLC [17,18]. It was shown, however, that the majority of patients on this initial treatment ultimately developed therapeutic resistance [19].

Osimertinib is an oral, third-generation EGFR-TKI recommended for use as a first-line therapy in adults with metastatic, EGFR-mutated NSCLC [12]. This was due to the results of the FLAURA study—a double-blind, phase 3 trial comparing first-line osimertinib to gefitinib or erlotinib in patients with EGFR-mutation-positive NSCLC—that osimertinib achieved first-line status and became listed as a priority recommendation by the National Comprehensive Cancer Network guidelines in 2019 [20,21]. Unlike prior EGFR-TKI generations, osimertinib selectively and irreversibly binds to EGFR-mutants inhibiting downstream signaling pathways and ultimately preventing tumor cell proliferation while promoting apoptosis [22]. Osimertinib has been shown to exert significant therapeutic results, especially in NSCLC patients with the T790M EGFR-associated mutation. The ADURA study revealed a 71% overall response rate (ORR) and 10.1-month progression-free survival (PFS) in T790M-mutant NSCLC patients [23]. It is important to note that when compared to first-generation TKIs, osimertinib shows a lower rate of grade 3 adverse events, indicative of an improved safety profile [20,24].

A 2020 systematic review and meta-analysis assessing the safety and efficacy of osimertinib in treating NSCLC based on overall survival (OS), PFS, treatment response, and adverse events (AEs), that included 47 studies, found osimertinib to be associated with an improved OS and PFS in all patients, including when compared to other EGFR-TKIs or chemotherapy. The incidence of severe AEs ranged from 0 to 5%, with the most common severe AE being pneumonia that occurred in 3% of patients [25]. Another retrospective multicenter study assessing the efficacy and safety of osimertinib in advanced or recurrent NSCLC at various treatment lines revealed a 26.8% incidence of adverse events of grade ≥ 3, with the most common being anemia and cutaneous toxicity (both at 5%). Neutropenia and grade 3 pneumonitis were observed in just a single patient each (2%) [26].

Previous research has categorized the tumor immune microenvironment (TIME) into three distinct phenotypes: inflamed, immune-excluded, and immune-desert. These phenotypes are known to impact the efficacy of immune checkpoint inhibitors (ICIs) in cancer treatment. Studies focusing on EGFR-positive tumors suggest that these tumors typically exhibit an uninflamed phenotype, which partly explains the limited effectiveness of ICIs in EGFR-positive patients [27,28,29].

Moreover, investigations into the tumor immune microenvironment during EGFR tyrosine kinase inhibitor (TKI) treatment have indicated that levels of immunosuppressive cells such as M2 macrophages and myeloid-derived suppressor cells (MDSCs) tend to increase following the development of resistance. Conversely, the levels of immune-active cells, including dendritic cells (DCs) and CD8+ T cells, often decrease after resistance emerges to EGFR-TKIs [29,30,31].

Osimertinib therapy may act as a bridge to allogeneic hematopoietic stem cell transplantation for patients with refractory acute myeloid leukemia. Interestingly, EGFR covalent inhibitors, like osimertinib, also show activity against stem and progenitor cells in chronic myeloid leukemia (CML). The introduction of imatinib and subsequent second-generation tyrosine kinase inhibitors (TKIs) has significantly transformed CML from a fatal disease into a manageable condition requiring lifelong therapy. Despite this progress, leukemia stem cells in CML can persist independently of BCR-ABL, leading to resistance to imatinib and presenting a major clinical challenge. Osimertinib’s selective cytotoxicity against CD34+ cells suggests that combining it with imatinib might offer a promising strategy to address BCR-ABL-independent mechanisms of resistance in CML [32,33,34].

Recently, it was reported that AML to anecdotal osimertinib monotherapy suggested significant antileukemia activity, particularly as these patients had experienced multiple rounds of chemotherapy or had a poor performance status precluding treatment with conventional induction regimens [32,35]. However, these findings are not directly applicable to our patient. Our patient had a very good performance status and initiated osimertinib therapy as a first-line treatment and has not undergone chemotherapy. Additionally, multiple bone marrow biopsies have been performed to rule out any second primary or hematologic malignancies.

**Figure 1:**
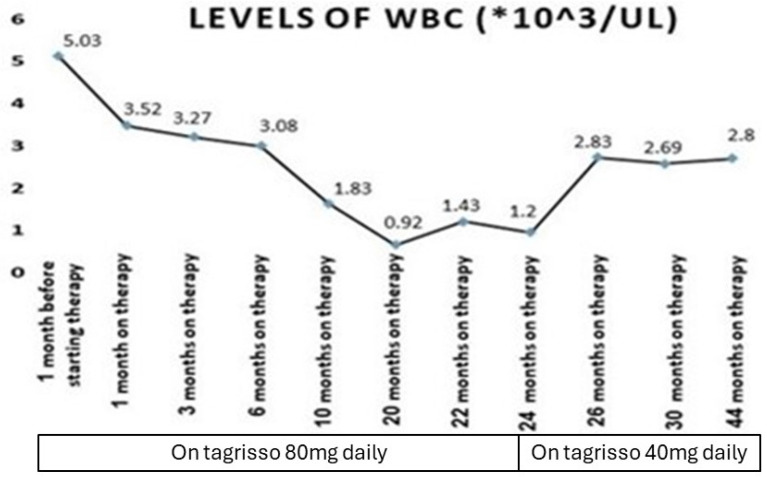
Timeline of lung cancer EGFR mutation treatment (Tagrisso): phases, dose changes, and their effects on leukocyte (WBC) levels during follow-up (*, multiplication).

**Figure 2:**
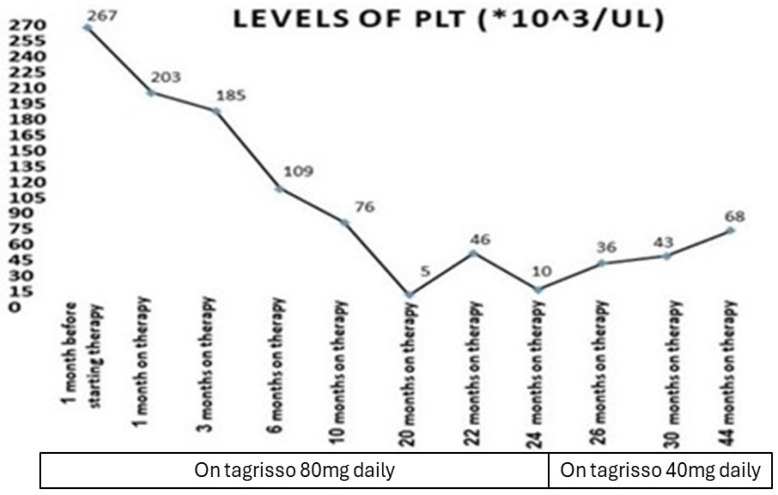
Timeline of lung cancer EGFR mutation treatment (Tagrisso): phases, dose changes, and their effects on platelet levels during follow-up (*, multiplication).

**Figure 3:**
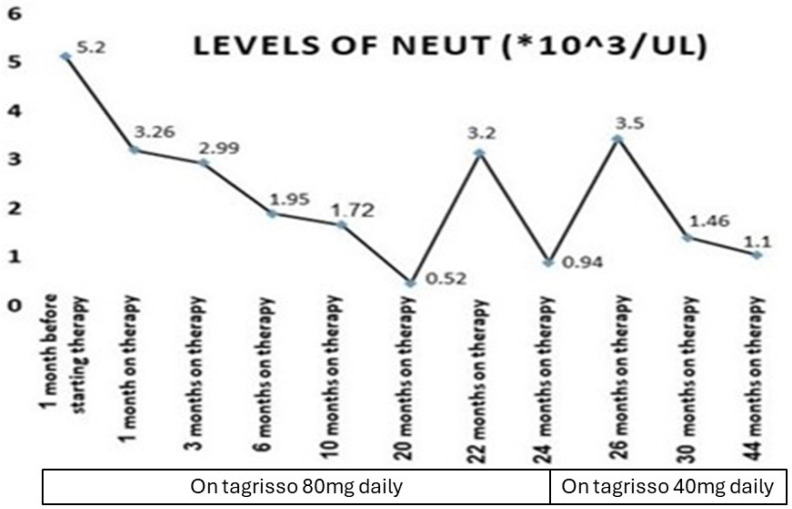
Timeline of lung cancer EGFR mutation treatment (Tagrisso): phases, dose changes, and their effects on neutrophil levels during follow-up (*, multiplication).

**Figure 4:**
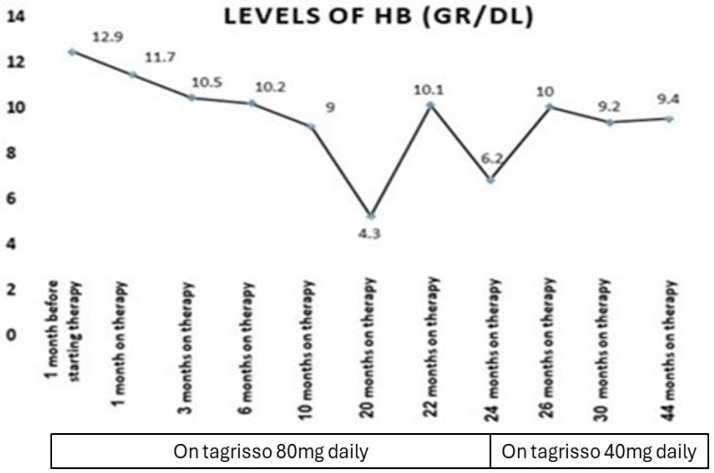
Timeline of lung cancer EGFR mutation treatment (Tagrisso): phases, dose changes, and their effects on hemoglobin levels during follow-up.

Our patient who we described before had a serious adverse event of myelosuppression during treatment with a third-generation epidermal growth factor receptor tyrosine kinase inhibitor (osimertinib) (Figure 1, Figure 2, Figure 3 and Figure 4). This is, in very rare cases, known to cause aplastic anemia, as described in the literature, and symptoms of drug-induced myelosuppression have been found to develop from days to months after initiation of treatment with the offending drug [36].

To the best of our knowledge, this is the first reported case of an EGFR-mutated NSCLC patient receiving first-line osimertinib suffering from life-threatening myelosuppression as a severe AE.

## 4. Conclusions

Herein, we report the rare occurrence of life-threatening, severe myelosuppression as a direct AE of first-line osimertinib treatment in an EGFR-mutated NSCLC patient showing good treatment response. Following temporary osimertinib discontinuation and concurrent blood transfusions to allow for toxicity recovery, a reduction in osimertinib dosage to 40 mg every second day was successful in reducing the myelosuppression to grade 1/2. Osimertinib may be safely administered to maintain stable disease in a patient with concurrent low-grade myelosuppression receiving blood transfusions as needed.

## Data Availability

The data either reside within this article itself or can be obtained from the authors upon making a reasonable request.
